# Reviewed article: Miyahara AK, Pepato PHM, Rocha JF, Oliveira AV, Maia JVB, Diniz V, Cerqueira BP, Hazin MAA, Canziani MEF, Goes MA. Prevalence of anemia in patients with chronic kidney disease attended at a nephrology outpatient clinic and its association with the outcome of renal replacement therapy over five years: a retrospective cohort study, Municipality of São Paulo, 2010-2018. Epidemiol Serv Saúde. 2026:35;e20250206

**DOI:** 10.1590/S2237-96222026v35e20250206.a

**Published:** 2026-03-09

**Authors:** Mariana Del Grossi Moura

**Affiliations:** 1 Instituto Sírio-Libanês de Ensino e Pesquisa, São Paulo, SP, Brazil Instituto Sírio-Libanês de Ensino e Pesquisa São Paulo SP Brazil

## First round comments

Prezados autores,

Agradecemos a submissão do manuscrito RESS-2025-0206, intitulado "O impacto da anemia em pacientes com doença renal crônica internados em um ambulatório de Nefrologia entre 2010 e 2018: um estudo de coorte retrospectivo sobre prevalência e evolução na cidade de São Paulo", à Revista Epidemiologia e

Serviços de Saúde: revista do SUS (RESS).

Após a revisão do seu artigo, identificamos a necessidade de revisões substanciais (Major Revision) para que o manuscrito esteja de acordo com os padrões editoriais da revista e para viabilizar sua publicação.

Reforço, a seguir, pontos que requerem atenção:

Sugiro repensar o título do manuscrito. O termo "impacto" costuma ser usado quando se pretende avaliar o efeito direto ou causal de uma intervenção, fator ou condição sobre um desfecho. Já o termo "associação" é mais apropriado em estudos observacionais, especialmente os transversais ou retrospectivos, onde se identifica uma relação estatística entre variáveis, sem que se possa afirmar causa e efeito;

Revisar o resumo com inclusão do delineamento do estudo, principais achados numéricos e conclusão compatível com os resultados;

O objetivo deve ser descrito ao final da introdução, em parágrafo corrido, com linguagem clara, alinhada ao tipo de estudo, e conectado à justificativa apresentada;

Em método, reorganizar o conteúdo conforme os itens do checklist STROBE e remover a seção "aspectos éticos" do corpo do texto, mantendo essa informação apenas na folha de rosto;

Na seção "Disponibilidade de dados", onde os dados analisados estão disponíveis, preferencialmente em repositório confiável como o SciELO Data, com link e DOI.

Em resultados, incluir o fluxograma da amostra, informar as perdas no seguimento e revisar siglas e legendas das tabelas e figuras;

Na discussão, evitar inferências causais, interpretar os achados com base em literatura adequada ao delineamento do estudo e apresentar as limitações de forma clara e objetiva.

As referências devem ser revisadas conforme as normas Vancouver, com links ativos e dados atualizados.

Reitero que todas as observações dos revisores devem ser atendidas de forma criteriosa e respondidas uma a uma em uma carta-resposta, justificando as alterações realizadas ou, quando pertinente, a manutenção da versão original. Apenas após essa reformulação, o manuscrito poderá ser reconsiderado para publicação.

Esta é uma decisão preliminar, e a nova versão poderá ser submetida a nova rodada de avaliação científica e editorial.

Recommendation: Major revision

## Second round comments

Prezados autores,

Agradecemos a submissão do manuscrito RESS-2025-0206.R1, intitulado "Frequência da anemia em pacientes com doença renal crônica admitidos em um ambulatório de nefrologia entre 2010 e 2018 e sua associação com o desfecho de terapia renal substitutiva em 5 anos: um estudo de coorte retrospectivo na cidade de São Paulo", à Revista Epidemiologia e Serviços de Saúde: revista do SUS (RESS).

Após a revisão do seu artigo, identificamos a necessidade de ajustes pontuais (Minor Revision) para que o manuscrito esteja de acordo com os padrões editoriais da revista e possa ser viabilizado para publicação.

Em especial, verificamos que algumas explicações fornecidas na carta-resposta anterior ainda não foram incorporadas ao texto do manuscrito:

Em resposta à pergunta do revisor - "Anemia de outras etiologias foi considerada um critério de exclusão.

Como ela foi diagnosticada? Em Resultados, nota-se que nenhum paciente foi excluído por essa razão. Favor esclarecer." - orientamos que a explicação apresentada seja incorporada diretamente ao texto do manuscrito, na seção de Métodos, item "Participantes".

Em resposta à pergunta do revisor - "Ausência de diagnóstico adequado de DRC também foi considerado um critério de exclusão. Favor definir o que é a 'ausência de diagnóstico adequado de DRC'." - orientamos que a explicação apresentada seja incorporada ao manuscrito, na seção de Métodos, item "Participantes".

Em resposta à pergunta do revisor - "Favor definir 'sobrevida renal'" - orienta-se que a definição fornecida na carta seja incorporada ao texto do manuscrito, na seção de Métodos, preferencialmente junto à descrição da análise de Kaplan-Meier.

Em resposta à pergunta do revisor - "Como os diagnósticos etiológicos da disfunção renal crônica foram firmados? Biópsia renal? Favor esclarecer." - orientamos que a explicação seja incorporada ao manuscrito, na seção de Métodos, item "Participantes" ou "Fonte de dados".

Em resposta à pergunta do revisor - "Qual o critério adotado no serviço para utilização de eritropoetina em pacientes com anemia, visto que apenas cerca de 14% deles receberam o medicamento?" - orientamos que a explicação apresentada seja incorporada ao texto do manuscrito, na seção de Métodos, item "Participantes" ou "Fonte de dados e variáveis".

Solicitamos que o(a) autor(a) envie uma nova carta-resposta, destacando de forma objetiva como cada uma dessas observações foi incorporada ao texto do manuscrito.

Após essa reformulação, o manuscrito poderá seguir para nova rodada de avaliação editorial e científica.

Recommendation: Minor revision.

